# The analgesic efficacy of erector spinae plane block versus paravertebral block in thoracic surgeries: a meta-analysis

**DOI:** 10.3389/fmed.2023.1208325

**Published:** 2023-08-17

**Authors:** Efrem Fenta, Simegnew Kibret, Metages Hunie, Tadese Tamire, Getachew Mekete, Abebe Tiruneh, Yewlsew Fentie, Kaletsidik Dessalegn, Diriba Teshome

**Affiliations:** Department of Anesthesia, College of Health Sciences, Debre Tabor University, Debre Tabor, Ethiopia

**Keywords:** acute postsurgical pain, erector spinae plane block, meta-analysis, paravertebral block, thoracic surgery

## Abstract

**Background:**

Acute postoperative pain after thoracic surgery might lead to chronic postsurgical pain (PSP), which lowers quality of life. The literature suggests thoracic paravertebral block (PVB) as a pain management approach. The ESPB (erector spinae plane block) is regarded to be an effective PVB alternative. The analgesic efficacy of the two analgesic therapies is controversial. The purpose of this study is to compare the analgesic efficacy of ESPB and PVB in preventing acute PSP.

**Methods:**

We searched relevant articles in PubMed, Cochrane Library, Embase, Web of Science, and Google Scholar databases. The primary outcome was postoperative pain score, with secondary outcomes including analgesic consumption, the frequency of rescue analgesia, and postoperative nausea and vomiting.

**Results:**

This meta-analysis included ten RCTs with a total of 670 patients. PVB significantly lowered the pain scores at movement at 12 h following surgery as compared to the ESPB. The PVB group used much less opioids within 24 h after surgery compared to the ESPB group. However, there were no significant differences between the groups in terms of postoperative rescue analgesia or in the incidence of postoperative nausea and vomiting (*p* > 0.05).

**Conclusion:**

PVB produced superior analgesia than ESPB in patients who underwent thoracic surgeries. In addition, PVB demonstrated greater opioid sparing effect by consuming much less opioids.

**Systematic review registration:**

This trial is registered on PROSPERO, number CRD42023412159.

## Introduction

Acute postoperative pain after thoracic surgery reduces quality of life and raises the possibility of chronic postsurgical pain (PSP) (1, 2). As a component of multimodal analgesia, regional anesthesia can efficiently manage pain, minimize the need for perioperative analgesic and anesthetic drugs, reduce postoperative nausea and vomiting, lower the risk of developing chronic pain, lower the incidence of postoperative respiratory complications, reduce the length of hospital stay, and increase patient satisfaction (3–7).

Thoracic paravertebral block (PVB) (8, 9), and erector spinae plane block (ESPB) (10–13) can be used for acute PSP management as a part of multimodal analgesia regimen for different types of procedures. Thoracic PVB is recommended by the enhanced recovery after surgery protocol as a trustworthy method for providing postoperative analgesia in thoracic procedures (14–16). However, it requires skilled hands due to its close proximity to the pleura, epidural space, and subarachnoid distance (6, 17).

The innovative interfacial plane block known as ESPB was initially introduced by Forero et al. in 2016 (18) and offers extensive analgesia in thoracic surgery. It can be used as a substitute for PVB because it is less intrusive, simpler, and safer to apply plane blocks that are applied in the plane of the spine’s erector muscles (1, 19). In this procedure, a local anesthetic solution is injected deeply into the erector spinae muscle, with an anticipated paravertebral distribution in both cranial and caudal directions (3, 20).

In thoracic PVB and ESPB, local anesthetics are injected into the costotransverse foramina, blocking the ventral and dorsal rami of the corresponding spinal nerves as well as sympathetic fibers, which causes sensory blockade over the anterolateral region of the thorax. The dermatomes covered by ESPB and PVB differ depending on the point of entrance, the amount, and the concentration of local anesthetics used (21, 22).

There is not a sufficient study comparing ESPB and TPVB. The few participants in the available studies contrasting ESPB with TPVB have yielded conflicting findings (12, 23, 24). Because ESPB is technically safe and simple, the hypothesis that it would be a better option to PVB is supported. Hence, we performed a meta-analysis to compare the analgesic effects of ESPB to the well-known thoracic PVB for patients who underwent thoracic surgery.

## Materials and methods

The Preferred Reporting Items for Systematic and Meta-analysis (PRISMA) is used to report this study. This review protocol has been registered in international prospective register of systemic review with registration number CRD42023412159.

### Search strategy

We searched relevant publications in the PubMed, Cochrane Library, Embase, Web of Science, and Google Scholar databases through April 2023. The search terms “Erector spinae plane block,” “Paravertebral block,” and “Thoracic surgery” were utilized.

### Inclusion/exclusion criteria

In this meta-analysis, patients (aged 18–81) who underwent thoracic surgery, randomized controlled trials, and studies comparing ESPB with PVB for postoperative analgesia were included in the study. Studies that compares PVB versus ESPB in combination with other blocks; Studies that compares PVB and ESPB combination; Studies that compares PVB versus ESPB for other procedures other than thoracic surgeries; and retrospective studies that compares PVB versus ESPB were excluded.

### Data extraction

Two authors looked through all of the article titles and abstracts to find publications that fulfilled the inclusion criteria. Studies with full paper copies were reviewed independently by two authors (Diriba Teshome and Simegnew Kibret), and choices on selection or rejection were made. A third reviewer settled any differences that might have arisen (Efrem Fenta). Names of authors, publication year, participant characteristics, sample size, block location, local anesthetic type and dose, operation type, duration of surgery, and study outcomes were retrieved from each included study (Table 1). The eligible publications were searched for raw data for continuous variables. If a variable’s range and median were provided in the full texts, the mean and standard deviation were then calculated from the range and median (34). If data values were presented graphically, WebPlotDigitizer was used to extract the numerical data (35). The primary outcome was the postoperative pain score, while the secondary outcomes were the consumption of analgesics used, the frequency of rescue analgesia, and postoperative nausea and vomiting at 24 h after surgery.

**Table 1:** Characteristics of included studies.

Author, year of publication	Characteristics of study participants(age, ASA)	Sample size (ESPB/PVB)	Location of block,	Local anesthetics(type, dose)	Surgery type	Duration of surgery (PVB & ESPB)	Outcome
Chen et al. (9), 2020	Patients aged 18–75 years, ASA I–II	24/24	PVB at T5-T7 ESPB at T5 level	20 mL of 0.375% ropivacaine for both blocks	VATS (Lobectomy, Segmentectomy, Wedge resection)	PVB = 128.4 (58.2), and 134.5 (43.1)	Cumulative morphine consumption, rescue analgesia, VAS pain scores at rest and while coughing at 0, 2, 4, 8, 24 and 48 h postoperatively.
Çiftçi et al. (25), 2020	Patients aged 18–65 years & ASA I-II	30/30	At the level of the T5 vertebra.	20 mL of 0.25% bupivacaine for both blocks	VATS (lobectomies/wedge resections)	PVB = 125.86 ± 17.67 min. & ESPB = 135.50 ± 29.13 min.	Total fentanyl consumption, rescue analgesia, VAS scores at 1, 2, 4, 8, 16, 24, 48 h at movement and at rest, Block procedure time, and side effects of the block (Nausea, Vomiting)
Duran et al. (26), 2022	Patients aged 18–75 years and ASA I-III	45/45	------------	-------------	Thoracotomy	---------	Morphine consumption
Fang et al. (27), 2019	Patients aged 18–81 years and ASA I-II	46/45	-------------	20 mL of 0.25% bupivacaine for either blocks	Thoracotomy (Wedge resection, Segmentectomy, Lobectomy)	72.61 ± 24.47 min and 78.33 ± 29.62 min.	VAS scores under the status of rest and cough at 1, 6, 12, and 24 h, puncture time and success rate of one puncture, and adverse effects (nausea and vomiting)
Jain et al. (28), 2022	Age ≥ 18 years, and ASA I-III	30/30	At T5/T6 level for PVB and T5 level for ESPB	20 mL 0.25% bupivacaine for either block	Thoracotomy, decortication, VATS, multiple open drainage system, and thoracomyoplasty	------------	Analgesic consumption, VAS scores at 0, 1, 3, 6, 12, and 24 h.
Taketa et al. (29), 2020	Patients aged 20–80 years, and ASA I-III	40/41	T4 or T5 intercostal level for both blocks	20 mL of 0.2% levobupivacaine for either block	VATS (radical lobectomy)	178.6 ± 28.2 and 179.3 ± 48.0	Rescue analgesia, NRS scores at rest and on movement at 0, 1, 3, 6, 12, and 24 h, and PONV.
Turhan et al. (30), 2021	Age ≥ 18 years, and ASA I-III	35/35	At the level of the T5 vertebra.	20 mL of 0.5% bupivacaine for either block	VATS	101.71 ± 24.55 min and 97.71 ± 43.05 min	Morphine consumption, VAS scores at rest and on movement, 0, 1, 4, 12, 24, 36 and 48.
Zengin et al. (31), 2022	Patients aged 18–80 years, and ASA I-III	30/30	At the level of the T5 vertebra.	20 mL 0.25% bupivacaine for either block	VATS (Wedge Resection, Segmenthectomy, Lobectomy)	175 (120–240) min and 150 (135–210) min.	Morphine consumption, rescue analgesia, Static and dynamic VAS resting and coughing scores at 1, 2, 4, 8, and 16 h, and PONV.
Zhang et al. (32), 2022	Patients aged 40–70 years, and ASA I-II	22/22	At T4 and T5 levels	30 mL of 0.5% ropiv- acaine	VATS lobectomy	126.05 ± 6.81 min and 126.82 ± 7.56 min.	VAS resting and coughing scores at 1, 6, 12, 24, and 48 h, PONV.
Zhao et al. (33), 2020	Patients aged 18–75 years, and ASA I-II	33/33	At T4 and T6 levels	30 mL 0.4% ropivacaine	VATS	107 ± 30 min and 121 ± 58 min.	Oxycodone consumption, VAS resting and coughing scores at 24 h, PONV.

### Evaluation of the risk of bias assessment

**Figure 1:**
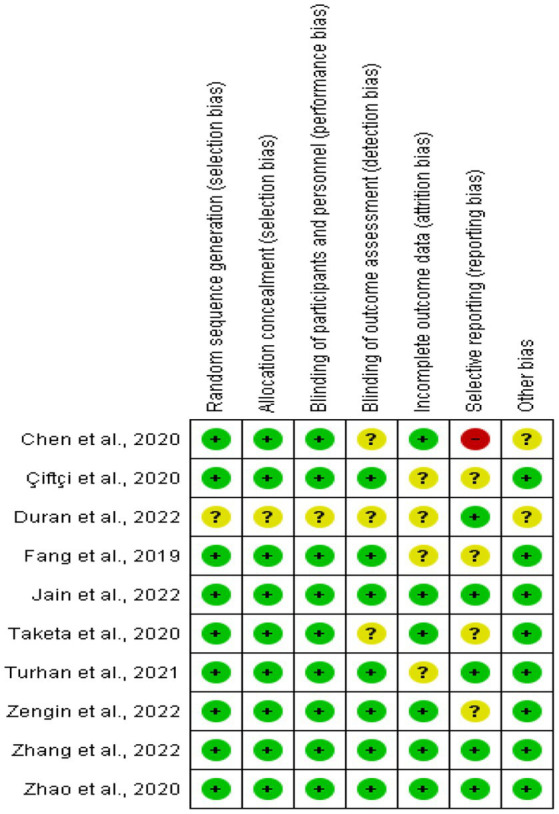
Risk of bias summary for each included study.

Using the Cochrane risk of bias tool, the risk of bias was evaluated and rated as low, unclear, or high independently by two researchers (Efrem Fenta and Tadese Tamire). Random sequence generation (selection bias), allocation concealment (selection bias), blinding of participants and personnel (performance bias), blinding of outcome assessment (detection bias), incomplete outcome data (attrition bias), selective reporting (reporting bias), and other bias were all taken into consideration when rating the included articles. A third reviewer (Diriba Teshome) resolved the differences that arose between the researchers. Figure 1 provides an overview of the risk of bias assessment.

### Statistical analysis

For this meta-analysis, Review Manager 5.4.1 (Cochrane Library, Oxford, UK) was utilized (Figure 1). A mean difference (MD) with a 95% confidence interval was reported for continuous data, including postoperative pain severity assessments and analgesic consumption at 24 h after surgery (CI). The relative risk (RR) at 95% CI was used to express the dichotomous data, such as the frequency of rescue analgesia and postoperative nausea and vomiting at 24 h after surgery. When the I^2^ was below 50%, a fixed-effect model was used, and when it was higher, a random-effect model was used. The funnel plot’s symmetry demonstrated that there was no publication bias.

## Results

### Characteristics of the included studies

**Figure 2:**
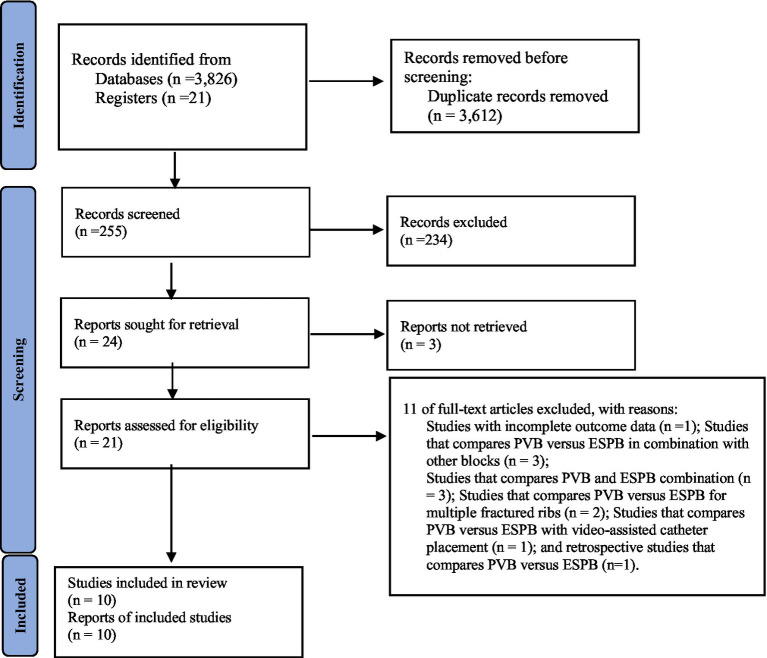
PRISMA 2020 flow diagram.

Our search parameters yielded a total of 3,847 studies, 3,612 of which were duplicates. Ten RCTs (9, 25–33) with a total of 670 patients (335 who received PVB, 335 who received ESPB) were included in this meta-analysis (Figure 2). Patients had thoracotomies in two trials (26, 27) and video-assisted thoracic surgery (VATS) in eight RCTs (9, 25, 28–33). Three trials (25, 30, 31) were carried out at the level of the T5 vertebra for both blocks, two trials (29, 32) at the T4 and T5 levels, and Zhao et al. (33) at the T4 and T6 levels. In a single trial by Chen et al. (9) PVB performed at T5 to T7 levels and ESPB at T5 vertebra levels, and in another trial by Jain et al. (28), PVB at T5 and T6 levels and ESPB at T5 levels were performed. But in two trials (26, 27), failed to mention where the blocks were performed (Table 1).

In terms of the local anesthetics type and dose used for both blocks, four trials (25, 27–29) used 20 mL of 0.25% bupivacaine, Zengin et al. (31) used 20 mL of 0.5% bupivacaine, Chen et al. (9) used 20 mL of 0.375% ropivacaine, Zhao et al. (33) used 30 mL of 0.4% ropivacaine, and Zhang et al. (32) used 30 mL of 0.5% ropivacaine for both PVB and ESPB. However, in a single trial (26), the dose of local anesthetics administered for both blocks was not reported (Table 1).

### Postoperative pain severity score at rest

**Figure 3:**
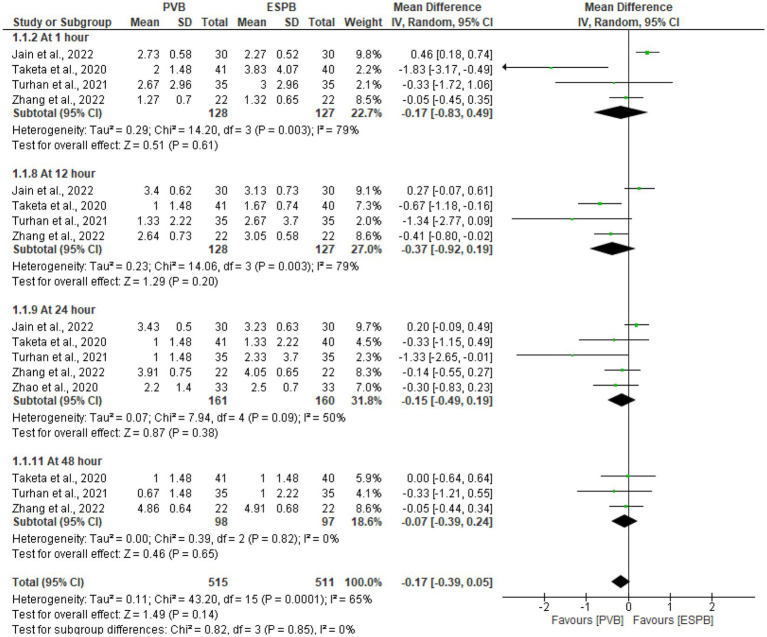
Forest plot of comparison: postoperative pain severity score at rest.

Following various thoracic procedures, the pooled analysis of postoperative pain scores at rest were conducted at 1 h (28–30, 32), 12 h (28–30, 32), 24 h (28–30, 32, 33), and 48 h (29, 30, 32). The pooled result demonstrated that, compared to the ESPB, PVB had comparable pain score at 24 h (MD −0.15 cm; 95% CI −0.49 to 0.19; P 0.38; *I*^2^ = 50%). There were no statistically significant differences reported in pain severity scores at a preset time points (Figure 3).

### Postoperative pain severity score at movement

**Figure 4:**
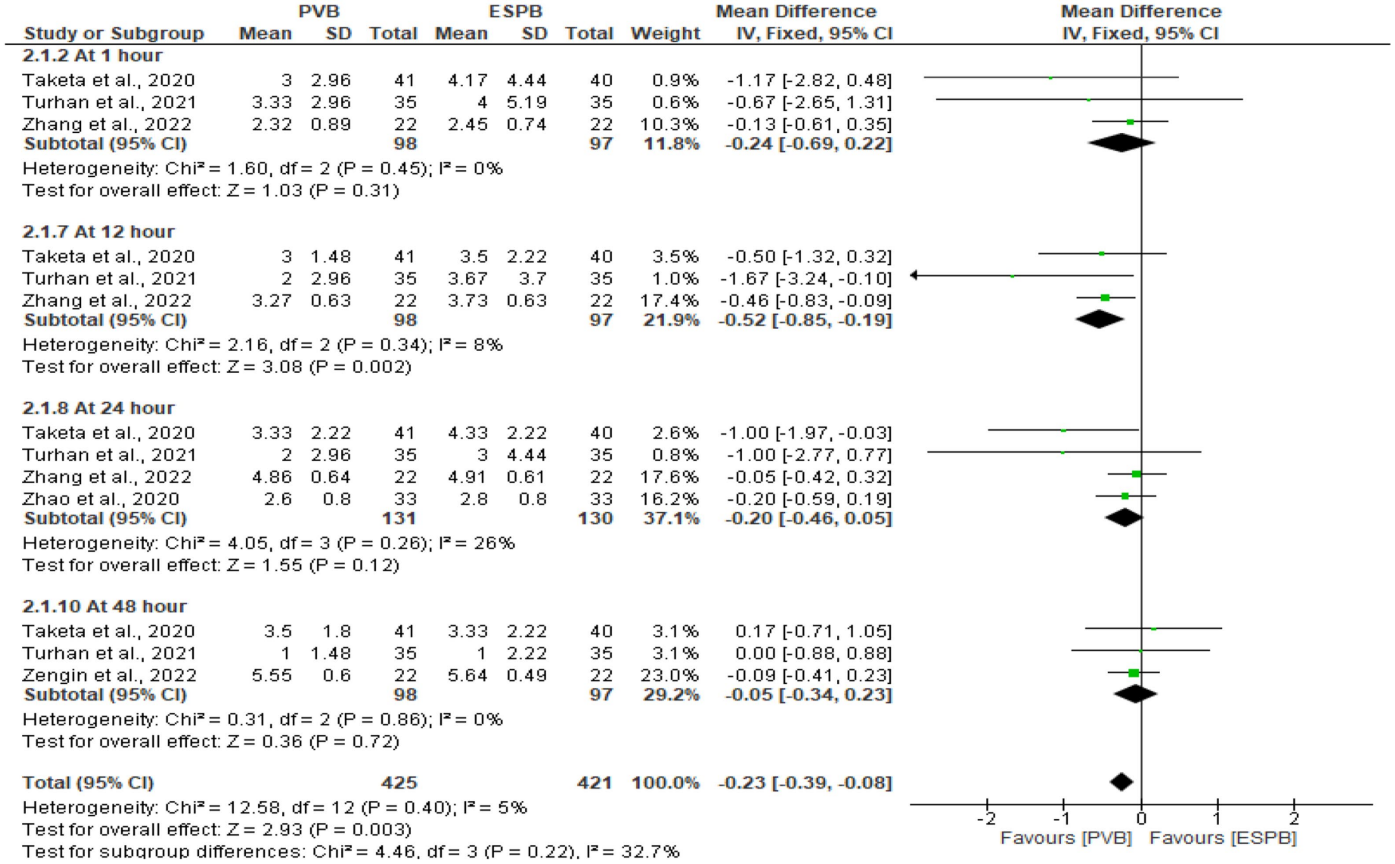
Forest plot of comparison: postoperative pain severity score at movement.

Following various thoracic procedures, the pooled analysis of postoperative pain scores at movement were conducted at 1 h (29, 30, 32), 12 h (29, 30, 32), 24 h (29, 30, 32, 33), and 48 h (29, 30, 32). The pooled results showed that PVB significantly lowered the pain score at 12 h (MD −0.52 cm; 95% CI −0.85 to −0.19; P 0.002; *I*^2^ = 8%). For other specified time points, were no statistically significant differences in the reported pain severity scores (Figure 4).

### Postoperative opioid consumption at 24 h (in morphine equivalents)

**Figure 5:**
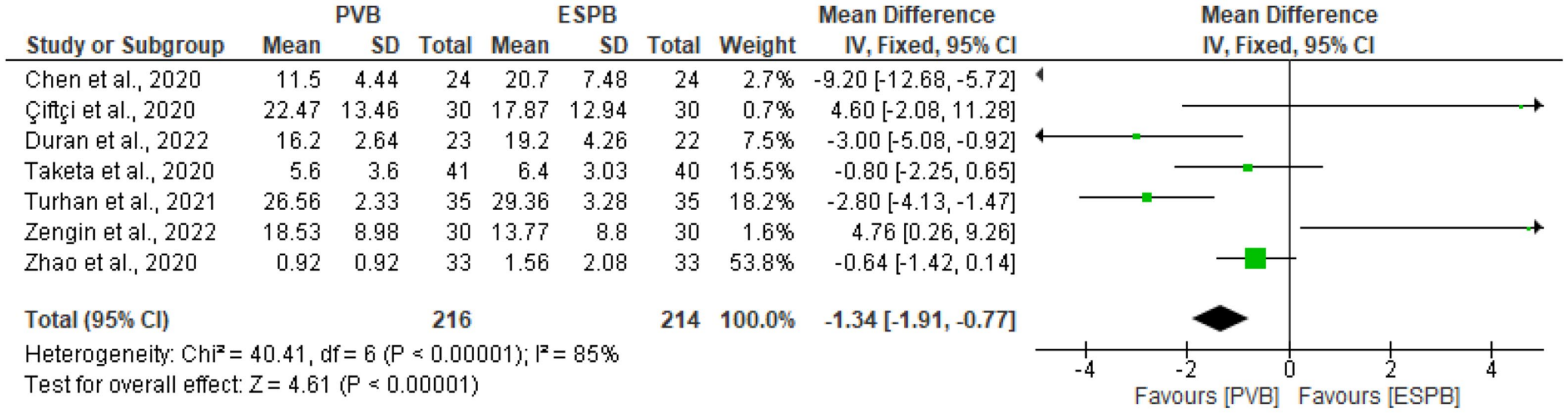
Forest plot of comparison: postoperative opioid consumption at 24 h (in morphine equivalents).

**Figure 6:**
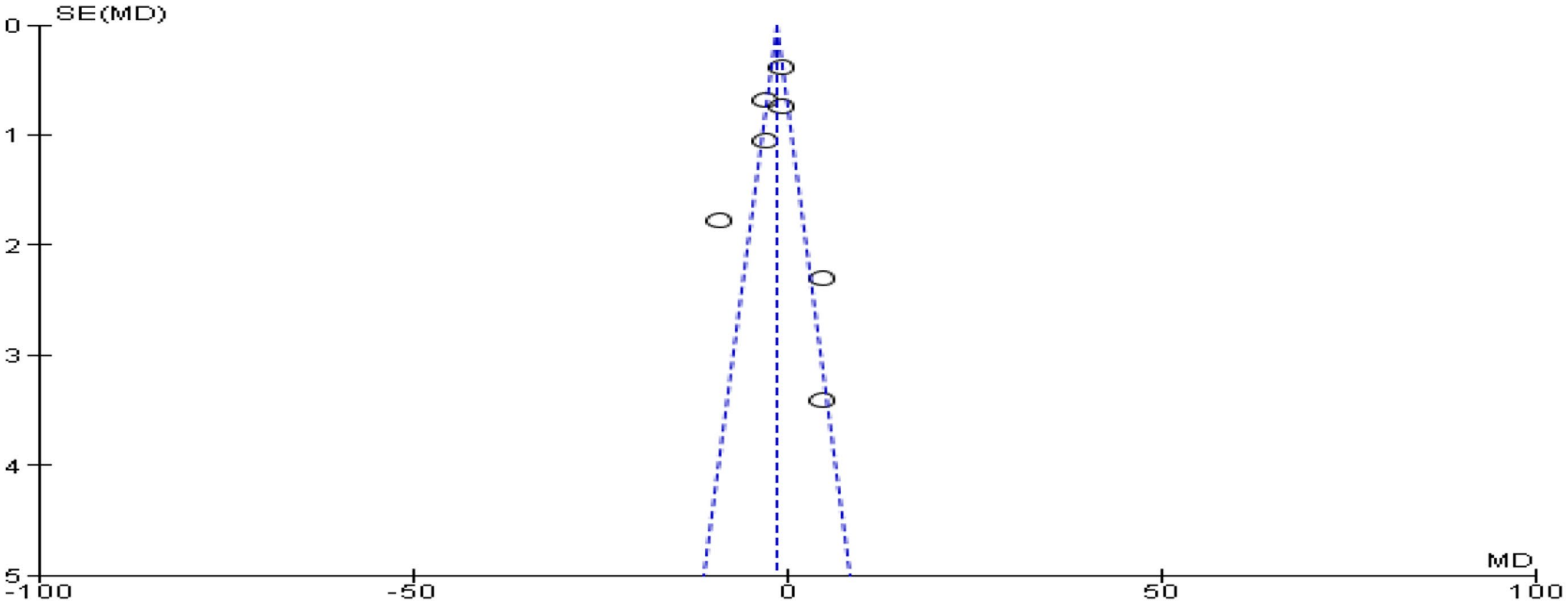
Funnel plot of comparison: postoperative opioid consumption within 24 h after surgery.

In seven trials (9, 25, 26, 29–31, 33) with 430 individuals, postoperative opioid consumption was reported. These included four trials (9, 26, 30, 31) had used morphine, one trial (33) had used oxycodone, and two trials (25, 29) had used fentanyl. Other opioids were converted into dosages of morphine equivalents to simplify data analysis. The findings of this meta-analysis revealed that the PVB group had significantly lower opioid use at 24 h (MD −1.34; 95% CI −1.91 to −0.77; *p* < 0.00001; *I*^2^ = 85%) following surgery than the ESPB group (Figure 5). The funnel plot’s symmetry demonstrated that there was no publication bias (Figure 6).

### Postoperative rescue analgesia within 24 h after surgery

**Figure 7:**
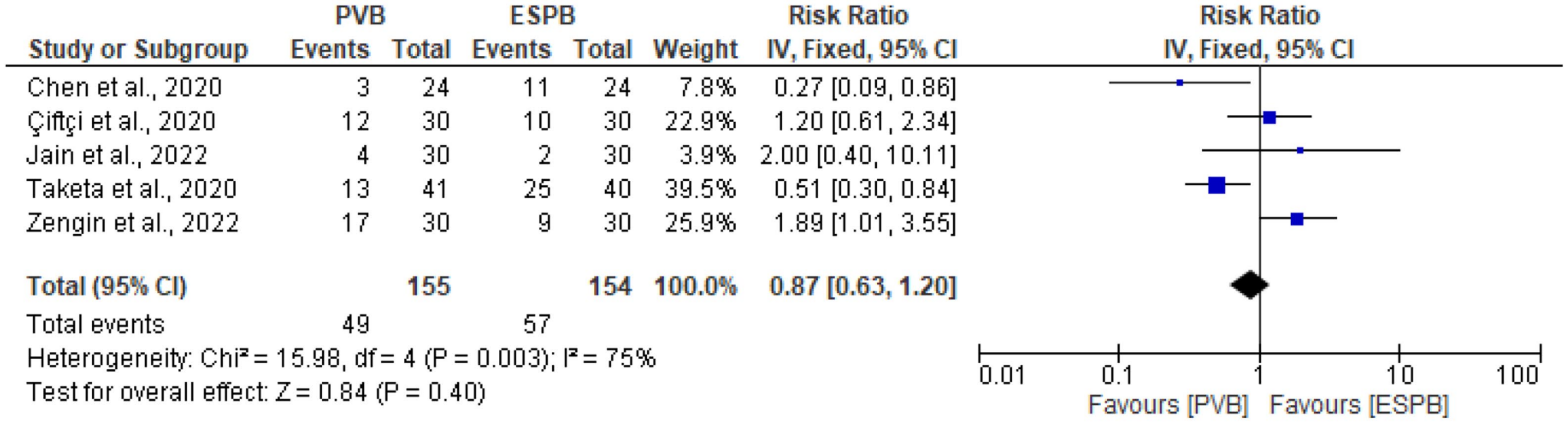
Forest plot of comparison: postoperative rescue analgesia within 24 h after surgery.

**Figure 8:**
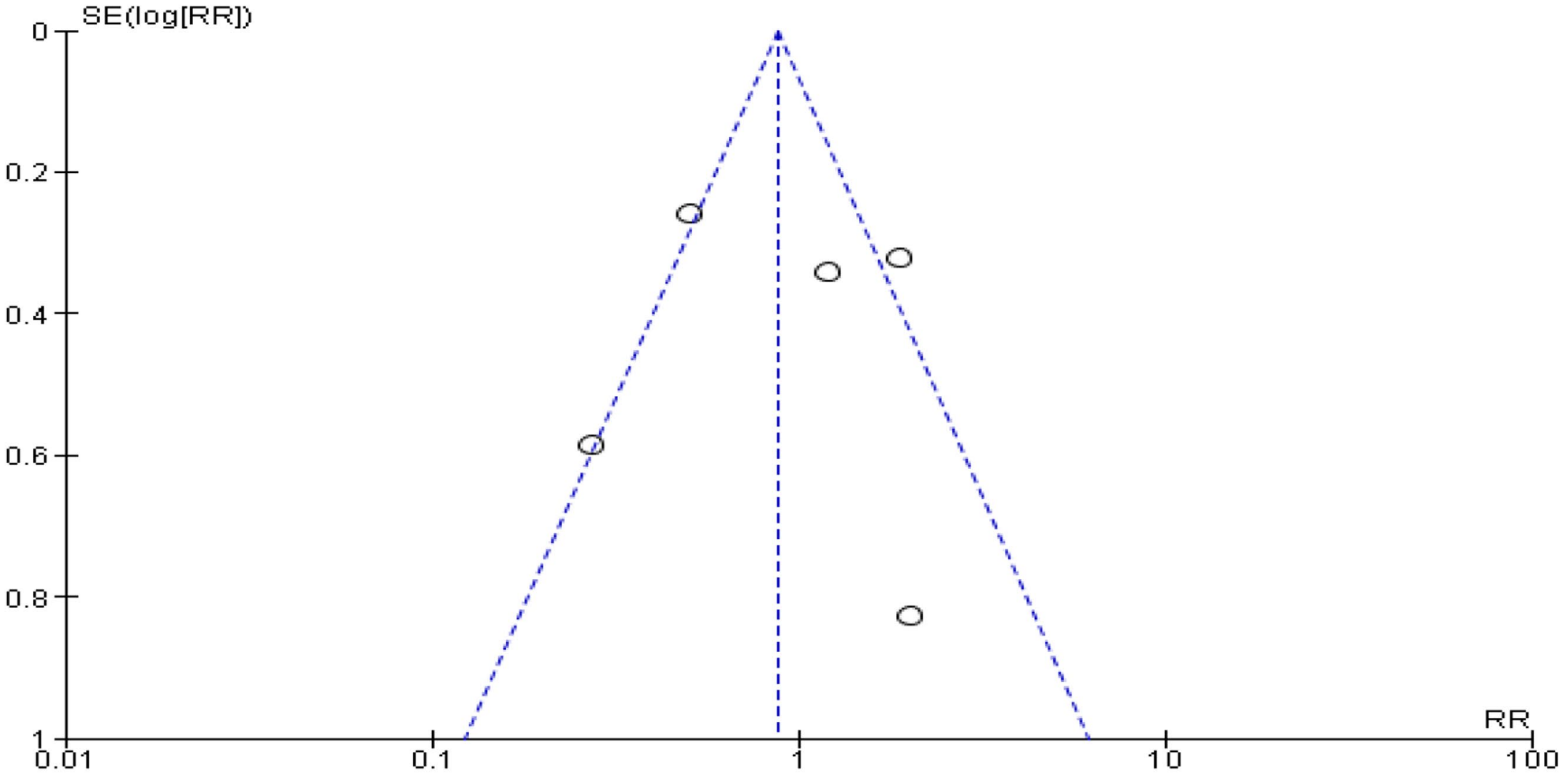
Funnel plot of comparison: postoperative rescue analgesia within 24 h after surgery.

Five trials (9, 25, 28, 29, 31) assessed the postoperative rescue analgesia within 24 h after surgery, and the results of this study showed no significant difference between the PVB and ESPB groups (RR 0.87, 95% CI 0.63 to 1.20, *p* = 0.40; *I*^2^ = 75%) (Figure 7). The symmetry of the funnel plot showed that there was no publication bias (Figure 8).

### The incidence of postoperative nausea and vomiting

**Figure 9:**
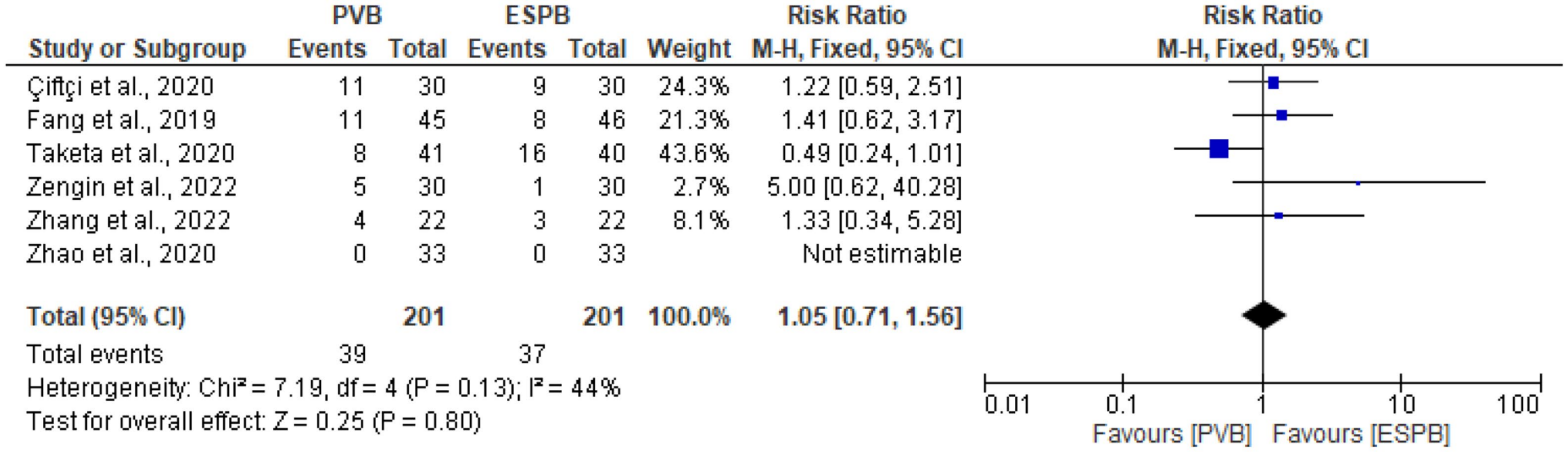
Forest plot of comparison: the incidence of postoperative nausea and vomiting.

The incidence of postoperative nausea and vomiting was reported in six trials (25, 27, 29, 31–33). The pooled result of this meta-analysis found that, there was no significant differences in the incidence of postoperative nausea and vomiting (RR 1.05, 95% CI 0.71 to 1.56, *p* = 0.80; I^2^ = 44%) between the PVB and ESPB groups (Figure 9).

## Discussion

The analgesic effectiveness of PVB over ESPB in patients following thoracic surgery was demonstrated by this meta-analysis, which included 10 RCTs comprising a total of 670 patients. PVB and ESPB have progressively substituted thoracic epidural analgesia, due to a variety of drawbacks, such as dural puncture, epidural hematoma, the increased risk of hypotension, and urine retention (9, 36). A systematic review and meta-analysis of 12 clinical trials involving a total of 541 patients revealed that thoracic PVB and thoracic epidural analgesia were comparable in terms of VAS scores at rest and during coughing at 4–8, 24–48 h postoperatively, but that the PVB group outperformed the epidural group significantly more effective at 48 at movement. Hypotension and urinary retention are more common in the group receiving epidural analgesia (37, 38). However, due to how close the paravertebral space is to the pleura, doing ultrasonography guided PVB still has a higher risk of pneumothorax. This risk is intensified by the fact that it frequently takes several thoracic injections to deliver optimal analgesia (39). The ESPB, potentially safer and need less technical expertise, is required to be used (40). The ESPB, a new interfacial plane block, significantly reduces pain after thoracic surgery (41, 42). It can be used as another PVB option with fewer complications because administering plane blocks in the plane of the erector spinae muscles is technically easy and safe (43). Using this method, a local anesthetic solution is deeply injected into the erector spinae muscle, with expected distributions to paravertebral space (44, 45). We conducted this meta-analysis to see whether it would be an appropriate substitute for PVB by considering ESPB’s ease of use and high success rate.

In this meta-analysis, we found that there were no statistically significant differences in pain scores at rest. The PVB significantly lowered the pain scores at movement at 12 h following surgery in contrast to the ESPB. For the other specified time points (1 h, 12 h, 24 h, and 48 h), there were no statistically significant differences in the reported pain severity scores during mobility. The findings of this meta-analysis also revealed that the PVB group used much less opioids within 24 h after surgery compared to the ESPB group. However, the results of this study showed no significant differences between the groups in terms of postoperative rescue analgesia within 24 h of surgery or in the frequency of postoperative nausea and vomiting.

Similar results were found in study by Xiong et al., who found that PVB significantly decreased pain scores at 0–1 h, 4–6 h, and 4 h at rest, significantly decreased pain scores at 4–6 h, 8–12 h, and 24 h at movement, and significantly decreased opioid consumption at 24 h post-op. However, there were no significant differences between the two groups in terms of the frequency of postoperative nausea and vomiting. In contrast to our findings, the incidence of rescue analgesia was significantly lower in the PVB group compared to the ESPB group after thoracic surgery (46). In line with our findings, a retrospective study by Sertcakacilar et al. showed that PVB caused superior analgesia than ESPB when comparing the efficiency of ultrasound-guided erector spinae plane block versus paravertebral block for postoperative pain relief in single-port VATS. Additionally, PVB demonstrated greater opioid sparing with significantly less opioids (19).

There are some limitation of this meta-analysis. The primary limitation of this meta-analysis could be the limited sample sizes for the publications included (ten papers) that were taken into account. More studies comparing PVB with ESPB for thoracic surgeries are also needed. The study may also have significant limitations due to clinical and methodological heterogeneity, such as variations in the block’s location, the dose and type of local anesthetics utilized during each procedure, and the types of thoracic surgeries performed.

## Conclusion

PVB provides a superior postoperative analgesia compared to ESPB as a part of multimodal analgesic regimen for patients undergoing thoracic surgeries. Additionally, by using significantly less opioids, PVB showed superior opioid sparing. Further research contrasting PVB with ESPB for thoracic procedures might be beneficial.

## Data availability statement

The original contributions presented in the study are included in the article/supplementary material, further inquiries can be directed to the corresponding author.

## Author contributions

EF, DT, SK, TT, and GM were developed the data and took part in the study’s design, carried out the statistical analysis, and drafted the manuscript. AT, MH, YF, and KD were collected, analyzed the data, and wrote the manuscript. All authors read and approved the manuscript.

## Conflict of interest

The authors declare that the research was conducted in the absence of any commercial or financial relationships that could be construed as a potential conflict of interest.

## Publisher’s note

All claims expressed in this article are solely those of the authors and do not necessarily represent those of their affiliated organizations, or those of the publisher, the editors and the reviewers. Any product that may be evaluated in this article, or claim that may be made by its manufacturer, is not guaranteed or endorsed by the publisher.

## Abbreviations

CI: Confidence interval
ERAS: Enhanced recovery after surgery
ESPB: Erector spinae plane; block
MD: Mean difference
PSP: Postsurgical pain
PVB: Paravertebral block
RCTs: Randomized controlled trials
RR: Relative risk
VATS: Video-assisted thoracoscopic surgery
